# Characterization of T cell activation and regulation in children with asymptomatic *Plasmodium falciparum* infection

**DOI:** 10.1186/s12936-018-2410-6

**Published:** 2018-07-13

**Authors:** Augustina Frimpong, Kwadwo Asamoah Kusi, Bernard Tornyigah, Michael Fokuo Ofori, Wilfred Ndifon

**Affiliations:** 1 West African Centre for Cell Biology of Infectious Pathogens (WACCBIP), Department of Biochemistry, Cell and Molecular Biology, University of Ghana, Legon, P. O. Box LG 54, Accra, Ghana; 2 Immunology Department, Noguchi Memorial Institute for Medical Research, College of Health Sciences, University of Ghana, P.O. Box LG 581, Accra, Ghana; 3 African Institute for Mathematical Sciences, P.O. Box DL 676, Cape-Coast, Ghana; 4 African Institute for Mathematical Sciences, University of Stellenbosch, 7 Melrose Rd, Muizenberg, Cape Town, 7945 South Africa

**Keywords:** Malaria, Regulatory T-cells, T-cell activation, Asymptomatic, Symptomatic, Children, *falciparum*, Immunity

## Abstract

**Background:**

Asymptomatic *Plasmodium* infections are characterized by the absence of clinical disease and the ability to restrict parasite replication. Increasing levels of regulatory T cells (Tregs) in *Plasmodium falciparum* infections have been associated with the risk of developing clinical disease, suggesting that individuals with asymptomatic infections may have reduced Treg frequency. However, the relationship between Tregs, cellular activation and parasite control in asymptomatic malaria remains unclear.

**Methods:**

In a cross-sectional study, the levels of Tregs and other T cell activation phenotypes were compared using flow cytometry in symptomatic, asymptomatic and uninfected children before and after stimulation with infected red blood cell lysates (iRBCs). In addition, the association between these T cell phenotypes and parasitaemia were investigated.

**Results:**

In children with asymptomatic infections, levels of Tregs and activated T cells were comparable to those in healthy controls but significantly lower than those in symptomatic children. After iRBC stimulation, levels of Tregs remained lower for asymptomatic versus symptomatic children. In contrast, levels of activated T cells were higher for asymptomatic children. Strikingly, the pre-stimulation levels of two T cell activation phenotypes (CD8+CD69+ and CD8+CD25+CD69+) and the post-stimulation levels of two regulatory phenotypes (CD4+CD25+Foxp3+ and CD8+CD25+Foxp3+) were significantly positively correlated with and explained 68% of the individual variation in parasitaemia. A machine-learning model based on levels of these four phenotypes accurately distinguished between asymptomatic and symptomatic children (sensitivity = 86%, specificity = 94%), suggesting that these phenotypes govern the observed variation in disease status.

**Conclusion:**

Compared to symptomatic *P. falciparum* infections, in children asymptomatic infections are characterized by lower levels of Tregs and activated T cells, which are associated with lower parasitaemia. The results indicate that T cell regulatory and activation phenotypes govern both parasitaemia and disease status in paediatric malaria in the studied sub-Saharan African population.

**Electronic supplementary material:**

The online version of this article (10.1186/s12936-018-2410-6) contains supplementary material, which is available to authorized users.

## Background

Falciparum malaria occurs when sporozoites inoculated into the human host develop in the liver into merozoites that infect red blood cells and cause clinical disease. The acquisition of natural immunity to falciparum malaria is slow and requires frequent exposure to the parasite over a period of time [1]. Despite previous exposure to the parasite, people in endemic areas may remain susceptible to clinical disease or they may be asymptomatic carriers of parasites as clinical immunity is only partial and never sterile. Also, repeated parasite exposure has been associated with limited protectiveness to vaccine candidates [2, 3]. The lack of a proper understanding of the immune responses occurring during natural infections has, for example, resulted in an inability to develop effective interventions such as vaccines.

Understanding the regulatory and protective immune responses during asymptomatic and clinical infections remain necessary to comprehend mechanisms that enable the control of infections as well as the persistence and survival of the parasite. A number of studies in malaria have associated protection from clinical disease with having a broad antibody repertoire [4–6]. Nonetheless, the presence of asymptomatic infections in children who may not have a broad antibody repertoire suggests that there is some level of immunity to the parasite by the host and this is characterized by the absence of clinical manifestations of the disease. Moreover, during *Plasmodium falciparum* infections, it is believed that the effector function of immune cells will be compromised due to immune regulation [7]. This may be induced by the specific expansion of certain T or B cell sub-sets and modulation of certain antigen presenting cells, such as the dendritic cells [8]. T cells express receptors that enable co-stimulation, activation, memory formation, and immune regulation to ensure effective and timely immune response induction upon antigen recognition. The expansion of specific cell sub-sets, especially those that express regulatory markers, may either enhance or inhibit the development of immunity against an infection. However, the association between such cellular activation and regulatory markers and parasite control during asymptomatic infections is inadequately understood.

Regulatory T cells are unique cell phenotypes that function to maintain homeostasis when the immune response is activated. The establishment of immune homeostasis may result in blocking the activity of other immune cells. For instance, CTLA-4 (also known as CD152), once activated, functions to inhibit activation of both antigen presenting cells and other T cells. Even though the role of Tregs during *P. falciparum* infections remains controversial, it has been observed that in both human and rodent malaria an early induction of Tregs may result in an increased parasite density [9–12]. Furthermore, the expansion of Tregs in malaria has been associated with decreased antigen-specific immune responses [11].

Also, a recent study by Kurup et al. [13] has shown that CTLA-4 Tregs expand during symptomatic malaria in both human and murine models, which is associated with decreased parasite clearance and impedes the acquisition of immunity in murine models. Other studies have also reported the upregulation of TNFRII on Tregs with asymptomatic parasitaemia [14]. There have also been reports on the upregulation of FOXP3 mRNA transcripts during acute malaria infections in children and naïve adults, which negatively correlated with Th1 memory responses [9, 15]. Nonetheless, other studies have also shown conflicting data whereby no association was found between the levels of Tregs and *Plasmodium* infection [16–19]. Collectively, these imply that the activity of Tregs associated with the development of protective immunity needs to be comprehended. The likely suggestions are that infections may cause the expansion of Tregs, which in turn may cause immune suppression and enhance parasite growth as observed in other studies [11, 20, 21].

This study aims to compare the expression levels of T cell activation and regulatory markers across symptomatic, asymptomatic and healthy control children living in hyperendemic areas with stable malaria transmission in Ghana. The Treg markers CD25+Foxp3+, the early activation marker CD69, and the late activation marker CD25 were measured. Tregs have an established role in suppressing effector immune responses to a variety of pathogens, including malarial parasites [22, 23]. CD69 expression in CD4+ T cells has been shown to correlate with the development of antigen-specific antibodies in experimental human falciparum malaria [24]. CD69 is a transmembrane glycoprotein expressed during early activation and increases with inflammation with the potential to induce cytotoxic activity once crosslinked [25–29], whereas CD25 (IL-2α receptor) has been associated with T cell proliferation and differentiation through the IL-2 cytokine [28]. This suggests that their combined expression may lead to an enhanced cellular immune activity. Therefore, it was hypothesized that asymptomatic infections have reduced Treg levels, such that exposure to *P. falciparum* is associated with increased cellular response and lower parasitaemia, which in turn feeds back to reduce cellular activation.

## Methods

### Study sites

Participants for the study were recruited from Asutsuare and Paakro sub-districts, which are hyperendemic for malaria transmission. Asutsuare has two malaria transmission seasons; June to August (high transmission season) and November to December (low transmission season) with an entomological inoculation rate of 14.6 infective bites/man/year whereas Paakro has May to June as the high transmission season and September to October as the low transmission season [30, 31]. Samples from participants were obtained during the high transmission seasons.

### Participants and sample collection

The study was approved by the Institutional Review Board of the Noguchi Memorial Institute for Medical Research, University of Ghana (Permit No. 096/15-16). A written informed consent was obtained from parents or guardians and assent appropriately received from the children before they were enrolled. Samples were obtained in a cross-sectional study from 57 children under 13 years old who satisfied the inclusion criteria with no known conditions that could interfere with the experiments. The participants were grouped into *P. falciparum*-infected asymptomatic children (n = 18), symptomatic malaria patients (n = 22) and healthy controls (n = 17). About 5 ml of venous blood was collected into heparin tubes before anti-malarial treatment. Both thick and thin smears were prepared and stained with Giemsa for parasite identification after screening for infection with rapid diagnostic tests. Haematological indices were determined by an automated haematological analyzer. PBMCs were isolated by ficoll gradient centrifugation and stored in liquid nitrogen until the time of the experiment. The PBMCs were cryopreserved in fetal bovine serum with 10% dimethyl sulfoxide (DMSO).

### Flow cytometry analysis

**Fig. 1:**
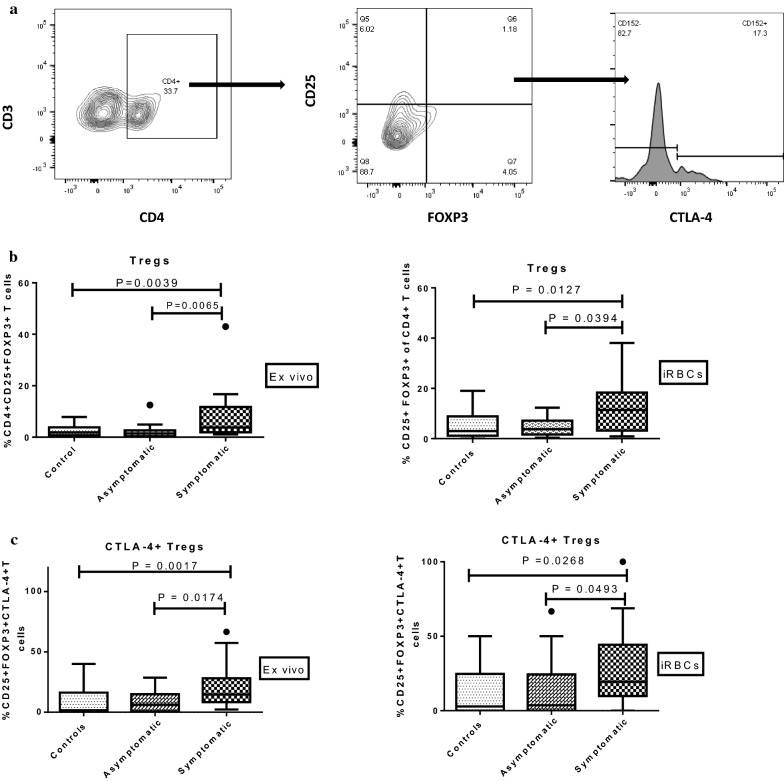
Percentage expression of CD4+CD25+FOXP3+ regulatory T cells among study groups. **a** Representative flow cytometry gating strategy for phenotyping CD4+CD25+FOXP3+ and CTLA-4+ Tregs in peripheral blood. The percentage expression and activation of regulatory T cells were analysed in healthy controls (n = 16), children with asymptomatic infections (n = 18) and symptomatic falciparum malaria (n = 22); for levels of **b** Tregs analysed as CD4+CD25+FOXP3+; and, **c** activated Tregs analysed as CD4+CD25+FOXP3+CTLA-4+ T cells both ex vivo and after iRBC stimulation. The data are presented as box plots with inter-quartile ranges. The 10th and 90th percentiles are denoted by whiskers. Medians are indicated by the horizontal lines across the boxes. Kruskal–Wallis test was used for comparisons, followed by Dunn’s test where necessary

**Fig. 2:**
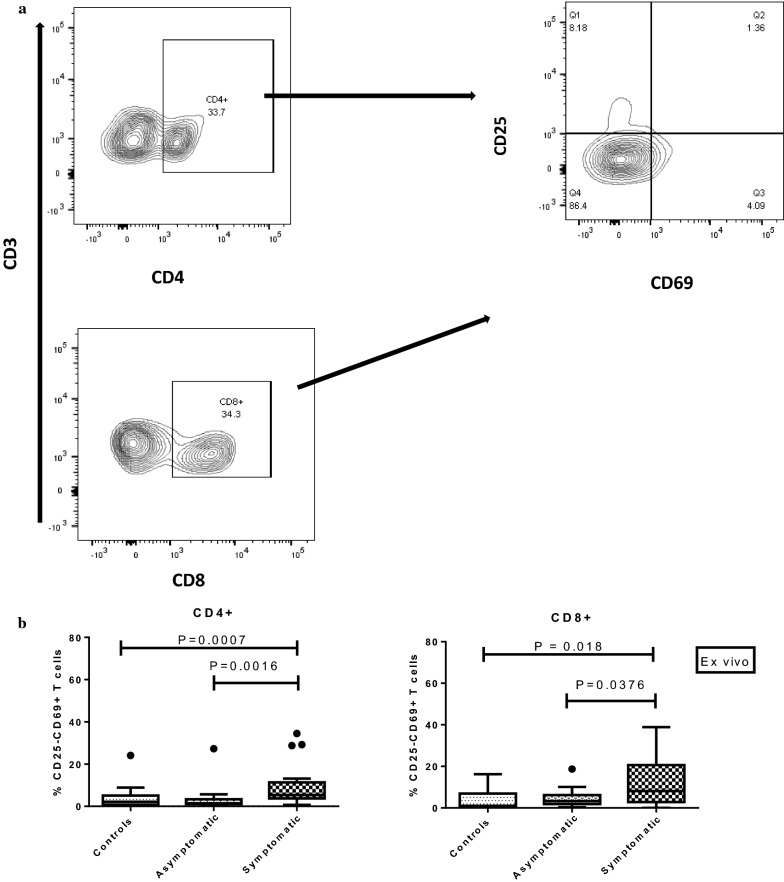
Expression of T-cell activation markers CD25/CD69 on T cells in PBMCs from the study cohort. **a** Representative flow cytometry gating strategy for phenotyping activation markers on CD4+ and CD8+ T cells ex vivo; the expression of the activation markers **b** CD25−CD69+ on T cells was analysed in healthy controls (n = 17), asymptomatic *P. falciparum*-infected children (n = 18), and symptomatic *P. falciparum*-infected children (n = 21). The data are presented as box plots with inter-quartile ranges. The 10th and 90th percentiles are presented as whiskers. Medians are indicated by the horizontal lines across the boxes. Kruskal–Wallis test was used for comparisons, followed by Dunn’s test where necessary

Stored PBMCs were thawed and washed. The viability was assessed by trypan blue dye exclusion method. Cells with viability greater than 95% were used in the assay. The cells were surface stained with the following antibodies for T cell sub-sets (anti-CD3, anti-CD4, anti-CD8), co-stimulation markers (anti-CD28, anti-CD57) and activation markers (anti-CD25, anti-CD69) (Additional file 1). The cells were washed, fixed and permeabilized using FOXP3 buffer set (BD) and intracellularly stained for regulatory markers Foxp3 (Biolegend) and CTLA-4 (BD). Fluorescence minus one controls and compensation were performed to set gates using single colour stained or unstained PBMCs. Data were compensated and analysed using Flowjo V10 software (Tree Star, San Carlos, CA, USA). The gating strategies are outlined in Figs. 1a and 2a.

### Stimulation of PBMCs with infected and uninfected red blood cells

*Plasmodium falciparum* parasites of the NF54 strain were cultured in O^+^ red blood cells at 3% haematocrit in culture medium (RPMI 1640 medium, 25 µg/ml of gentamycin, 10% heat-inactivated O^+^ human serum). The culturing was done in the presence of 7.5% sodium bicarbonate at 37 °C in a 5% O_2_, 5% CO_2_ and 90% N_2_ atmosphere. PBMCs were later thawed and rested for 6 h in 10% fetal bovine serum. About 400,000 cells were later stimulated with intact *P. falciparum* trophozoites/schizont (NF54 clone)-infected RBCs (iRBCs; 3 iRBCs: 1 PBMC) or uninfected RBCs (uRBCs; 3 uRBCs: 1 PBMC) and cultured in complete RPMI 1640 in 5% CO_2_ at 37 °C. After 4 h of stimulation, brefeldin A was added at a concentration of 10 µg/ml. Cells were washed and stained after 18 h with the following monoclonal antibodies; anti-CD3 (APC-H7), CD4 (BUV 395), CD8 (PerCP-Cy5.5), CD25 (PE-CF594), CD69 (PE-Cy7), CD152/CTLA-4 (APC), FOXP3 (PE) all from BD. The cells were washed by centrifugation before staining for both extracellular and intracellular markers.

### Statistical analysis

Data analyses were performed with the GraphPad Prism version 6.01 (GraphPad Software, Inc.) and the R statistical software version 3.4.0 (R Foundation for Statistical Computing). The demographics and clinical characteristics of the study participants were compared among the 3 study groups using Chi square test for categorical variables, Kruskal–Wallis or One-way ANOVA for continuous variables, Mann–Whitney U test and Wilcoxon-Signed Rank Test for paired comparisons for data that were not normally distributed. For comparing the markers of T cells among the 3 study populations, the Kruskal–Wallis test was used with a Dunn’s post hoc test or a Bonferroni correction for multiple comparisons where necessary. Spearman’s rank correlation was used to determine associations between markers. Support vector machine model, a supervised machine-learning algorithm was used to predict disease status. Statistical significance was set at P-values < 0.05.

## Results

### Characteristics of the study population

Venous blood samples were obtained from 57 children including 18 with asymptomatic *P. falciparum* infections, 22 with symptomatic malaria, and 17 with no *P. falciparum* parasites detected in blood by microscopy or rapid diagnostic test (Table 1). There was no statistically significant difference between the ages of children in the asymptomatic versus symptomatic groups. In contrast, the healthy controls were significantly older than the asymptomatic (*P *= 0.0404) and symptomatic children (*P *= 0.0123). Also, the mean haemoglobin levels in asymptomatic children were significantly higher compared to the symptomatic children (*P *= 0.0348). However, levels were comparable between the children in the control group and asymptomatic or symptomatic groups. Total leukocyte counts were significantly higher in the asymptomatic children compared to symptomatic children (*P *= 0.0478) but comparable to controls. Even though the median lymphocyte counts did not differ significantly amongst the groups, lower levels were found in the symptomatic group than in the asymptomatic and control groups. Also, platelet levels decreased with the severity of *P. falciparum* infections. The median platelet counts in the symptomatic children were significantly lower than in the asymptomatic (*P *= 0.0029) and control (*P *< 0.0001) groups. Children in the asymptomatic group had statistically similar platelet counts as the control group. Parasitaemia levels were significantly lower in asymptomatic children compared to children with symptomatic infection (*P *= 0.0009).

**Table 1:** Demographics and clinical characteristics of the study participants

Characteristics	Control	Asymptomatic	Symptomatic	P values
Sample size	n = 17	n = 18	n = 22	
Age (IQR), years	9 (8–11)	7 (4.5–9)	6 (4.8–7)	0.0087^a^
Female (%)	52.94	44.44	50	0.8765^b^
Mean haemoglobin (IQR), g/dl	11.5 (10.8–12.1)	12.7 (11.7–13.58)	10.7 (8.8–13.1)	0.0402^c^
Parasitaemia (IQR), µl	NA	845 (260.7–3812)	13,973 (7238–58,764)	0.0009^d^
Leukocytes (10^9^/l)	7 (5.7–8.0)	7.7 (6.1–9.6)	5.1 (1.2–8.3)	0.0436^a^
Lymphocytes (10^6^/l)	2.9 (2.5–3.6)	2.1 (1.2–3.45)	1.9 (1.3–3.9)	0.0889^a^
Platelets (10^9^/l)	305 (237–356)	223 (193–280)	101 (61–198)	> 0.0001^a^

*IQR* interquartile range, *NA* not applicable

^a^Kruskal–Wallis test

^b^Chi square test

^c^One-way ANOVA

^d^Mann–Whitney U test

### Decreased levels of regulatory T cells in asymptomatic *Plasmodium falciparum* infections

To investigate if there are any differences between the *P. falciparum*-infected group and healthy controls with respect to T cell regulation, the levels of Treg populations in the 3 study groups were determined. Here, Treg populations were classified as CD3+, CD4+, CD25+ and FoxP3+ (Fig. 1a).

For the peripheral blood mononuclear cells (PBMCs) analysed directly without stimulation (ex vivo), it was observed that Tregs had a lower frequency in the asymptomatic children compared to the symptomatic children (*P *= 0.0065), but levels were comparable between asymptomatic and control groups (*P *> 0.05). Also, the Treg frequency in the symptomatic children was higher than in the controls (*P *= 0.0209, Fig. 1b). This trend remained the same after the cells were stimulated with iRBCs in vitro; lower levels of Tregs were found in the asymptomatic children than in the symptomatic children (*P *= 0.0394), while levels were comparable to the controls. Similarly, levels in the symptomatic children were higher than in the controls (*P *= 0.0391, Fig. 1b).

Furthermore, the levels of activated Tregs based on the expression of CTLA4, an immunosuppressive marker that inhibits activation of immune cells by direct contact, were determined. The levels of CTLA4+ Tregs differed significantly across the study populations (*P *= 0.0017). The levels of CTLA4+ Tregs in children with asymptomatic malaria were significantly lower than in those with clinical malaria (*P *= 0.0174, Fig. 1c) but comparable with the control group. However, levels of CTLA4+ Tregs in the symptomatic group were significantly higher than in the control group (*P *= 0.0034). In addition, after iRBC stimulation, CTLA-4+ Treg levels remained significantly lower in the asymptomatic group compared to the symptomatic group (*P *= 0.0493) but were comparable to levels observed in healthy controls (*P *= 0.5457, Fig. 1c).

### Decreased expression of CD69 activation marker on T cells in asymptomatic *Plasmodium falciparum* infections before infected red blood cell stimulation

With the observed levels of Tregs being lower in the asymptomatic children compared to symptomatic children, the extent of cellular activation was measured to determine if they may differ across the study groups. The expression of the CD69 activation marker on both CD4+ and CD8+ T cell sub-sets before in vitro stimulation was investigated (Fig. 2a). Children with asymptomatic malaria had significantly lower levels of CD69+ expression on CD4+ T cells compared to children with symptomatic disease (*P *= 0.0016) but had comparable levels with the controls (*P *> 0.05, Fig. 2b). In addition, children with symptomatic malaria had a higher level of the CD4+CD69+ T cells than the controls (*P *= 0.0068, Fig. 2b). This trend was the same for the CD8+CD69+ T cells.

### The pattern of expression of CD25 on T cells among symptomatic, asymptomatic *Plasmodium falciparum* infections and healthy controls before infected red blood cell stimulation

**Fig. 3:**
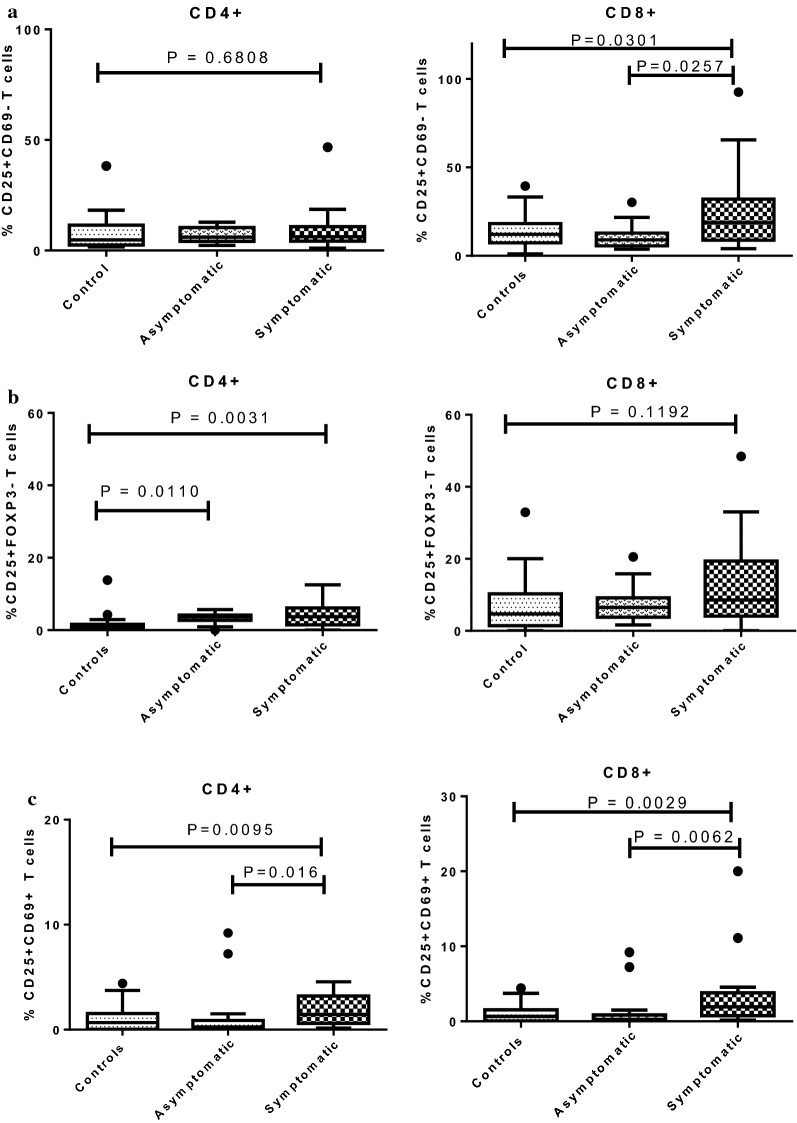
Expression of T-cell activation markers CD25/CD69 on T cells in PBMCs from the study cohort. **a** CD25+CD69−; **b** CD25+FOXP3− cells; and, **c** CD25+CD69+ on T cells was analysed in healthy controls (n = 17), asymptomatic *P. falciparum*-infected children (n = 18), and symptomatic *P. falciparum*-infected children (n = 21). The data are presented as box plots with inter-quartile ranges. The 10th and 90th percentiles are presented as whiskers. Medians are indicated by the horizontal lines across the boxes. Kruskal–Wallis test was used for comparisons, followed by Dunn’s test where necessary

The expression levels of CD25, a late activation marker, on CD4+ and CD8+ T cells were determined (Fig. 2a). Except for CD8+CD25+CD69− T cells, levels of CD25 were not significantly different between asymptomatic and symptomatic children. There was no significant difference in the expression of CD25 on CD4+ T cells across the study populations (*P *= 0.4971, Fig. 3a). However, for CD8+ T cells, the expression of CD25 was significantly lower in the asymptomatic group compared to the symptomatic children (*P *= 0.0257), whereas levels between the asymptomatic and control groups were comparable (Fig. 3a).

CD25+FOXP3− T cells have been classified as activated effector Th 1 cells capable of secreting effector cytokines, such as IFNγ (interferon gamma), TNF (tumour necrosis factor) and IL-10 (interleukin-10). Therefore, the ex vivo expression of these markers was compared across the study groups. No significant difference was observed in the expression of CD25+FOXP3− on either CD4+ or CD8+ T cell sub-sets between asymptomatic and symptomatic children. However, the levels of CD25+FOXP3− on CD4+ T cells were significantly lower in the healthy controls when compared to the asymptomatic (*P *= 0.0063) or symptomatic children (*P *= 0.0024). In addition, no significant difference was observed in the expression of CD8+CD25+FOXP3− T cells across the study groups (*P *= 0.1192, Fig. 3b).

### Decreased expression of CD25+CD69+ T cell activation markers during asymptomatic *Plasmodium falciparum* infections before infected red blood cell stimulation

The expression of CD69 activation marker on both CD4+ and CD8+ T cell subsets before in vitro stimulation was investigated in the three study groups (Fig. 2a). For the PBMCs analysed ex vivo, CD4+ and CD8+ T cells expressing both CD25 and CD69 were higher in the symptomatic when compared to the asymptomatic group (*P *= 0.016 and *P *= 0.0062) and healthy controls (*P *= 0.047 and *P *= 0.0232), respectively (Fig. 3c). However, no significant difference was observed between the asymptomatic group and controls (*P *> 0.05) for both T cell sub-sets (Fig. 3c).

### Increased expression of activation marker on T cells in asymptomatic infections after infected red blood cell stimulation

**Fig. 4:**
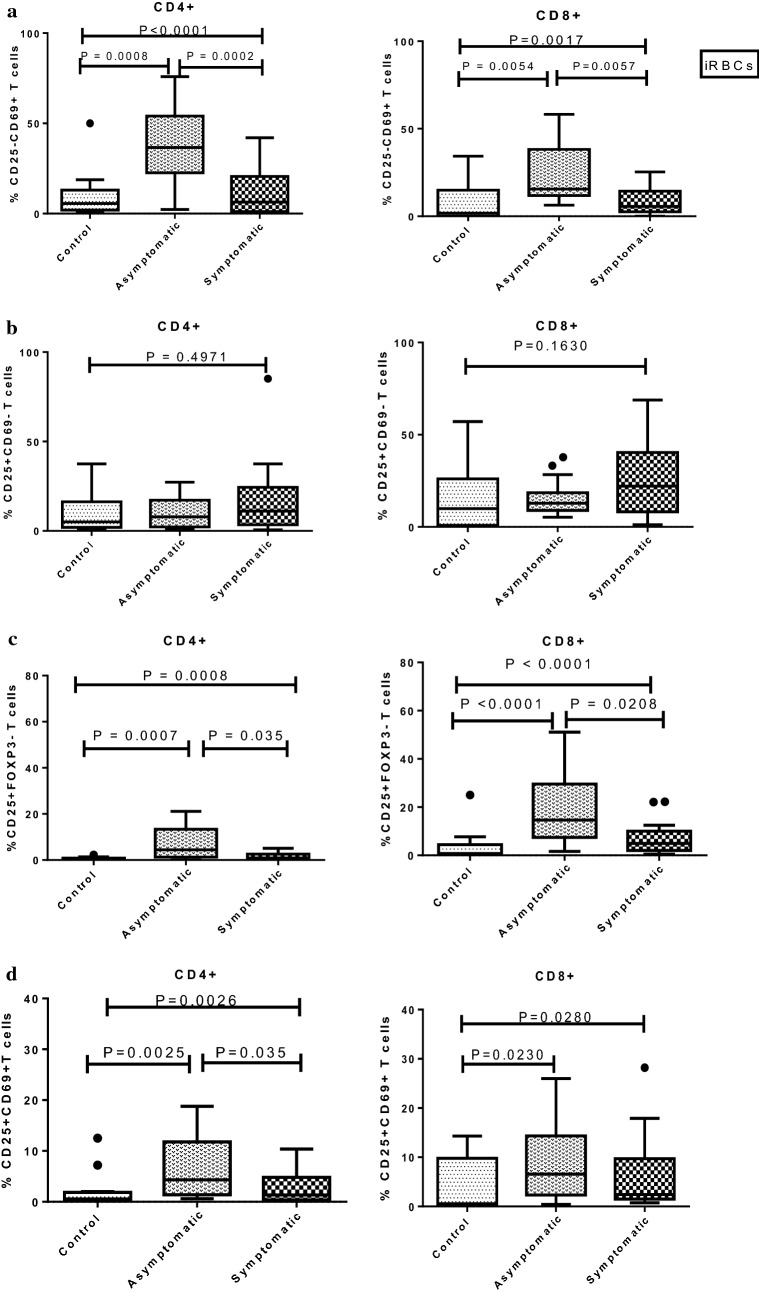
Expression of activation markers CD25/CD69 on T-cells from the study cohorts after iRBC stimulation. PBMCs were stimulated with iRBC lysates (iRBCs) to determine the levels of activation markers (CD25/CD69) on both CD4+ and CD8+ T cell sub-sets. The percentage expression of **a** CD25−CD69+; **b** CD25+CD69−; **c** CD25+FOXP3−; and, **d** CD25+CD69+ T cells was analysed in healthy controls (n = 17), asymptomatic *P. falciparum*-infected children (n = 18) and symptomatic *P. falciparum*-infected children (n = 21). The data are presented as box plots with inter-quartile ranges. The 10th and 90th percentiles presented as whiskers. Medians are indicated by the horizontal lines across the boxes. The Kruskal–Wallis test was used for statistical comparisons between groups. P values < 0.05 were considered to be significant after Dunn’s test to correct for multiple comparisons

When PBMCs were stimulated in vitro with iRBCs, levels of activated CD4+CD69+ T cells in the asymptomatic children increased significantly above those found in the symptomatic children (*P *= 0.0002) and controls (*P *= 0.0008, Fig. 4a). Levels of CD4+CD69+ T cells did not differ significantly between symptomatic children and controls. Higher expression of CD8+CD69+ cells was also observed in the asymptomatic cohort compared to both symptomatic children (*P *= 0.0057) and controls (P = 0.0054) (Fig. 4a). As was observed in the CD4+ T cell compartment, levels of CD8+CD69+ T cells did not differ significantly between symptomatic children and controls (Fig. 4a).

Also, no significant difference was found in the expression of CD25+CD69− T cells on any of the T cell sub-sets across the study groups (Fig. 4b). Levels of CD25+FOXP3− activated effector T cells were increased in the asymptomatic children compared to symptomatic and healthy controls (Fig. 4c). Increased levels of the CD4+CD25+FOXP3− activation marker were found in the asymptomatic group compared to the symptomatic (*P *= 0.035) and control (*P *= 0.0007) groups. Likewise, CD8+CD25+FOXP3− activated T cells were significantly increased in the asymptomatic group compared to the symptomatic (*P *= 0.0208) and control (*P *< 0.0001) groups (Fig. 4c). In addition, a significant increase in levels of double-positive CD4+CD25+CD69+ T cells were observed in the asymptomatic children compared to the symptomatic children (*P *= 0.035) and controls (*P *= 0.0025, Fig. 4d). Likewise, higher expression of CD8+CD25+CD69+ cells was observed in the asymptomatic cohort when compared to the healthy controls (*P *= 0.023) but not the symptomatic children (*P *> 0.05, Fig. 4d).

### Cellular activation and Treg frequency govern parasitaemia and disease status

Correlations between the 24 considered T cell phenotypes (including the pre- and post-iRBC stimulation levels of Tregs) and parasite control (as measured by parasitaemia levels) were investigated. After applying a Bonferroni correction for multiple comparisons, significant positive correlations were found between parasitaemia and the levels of both CD8+CD69+ (r = 0.4128658, P = 0.0016) and CD8+CD25+CD69+ (*r *= 0.4070214, *P *= 0.0018) T cells measured before iRBC stimulation, and the levels of both CD4+CD25+Foxp3+ (*r *= 0.4772815, *P *= 0.0002) and CD8+CD25+Foxp3+ (*r *= 0.4772714, *P *= 0.0003) T cells measured after stimulation. Strikingly, levels of these four T cell phenotypes together accounted for 68% of the variation in parasitaemia observed in asymptomatic and symptomatic children (Additional files 2, 3).

**Fig. 5:**
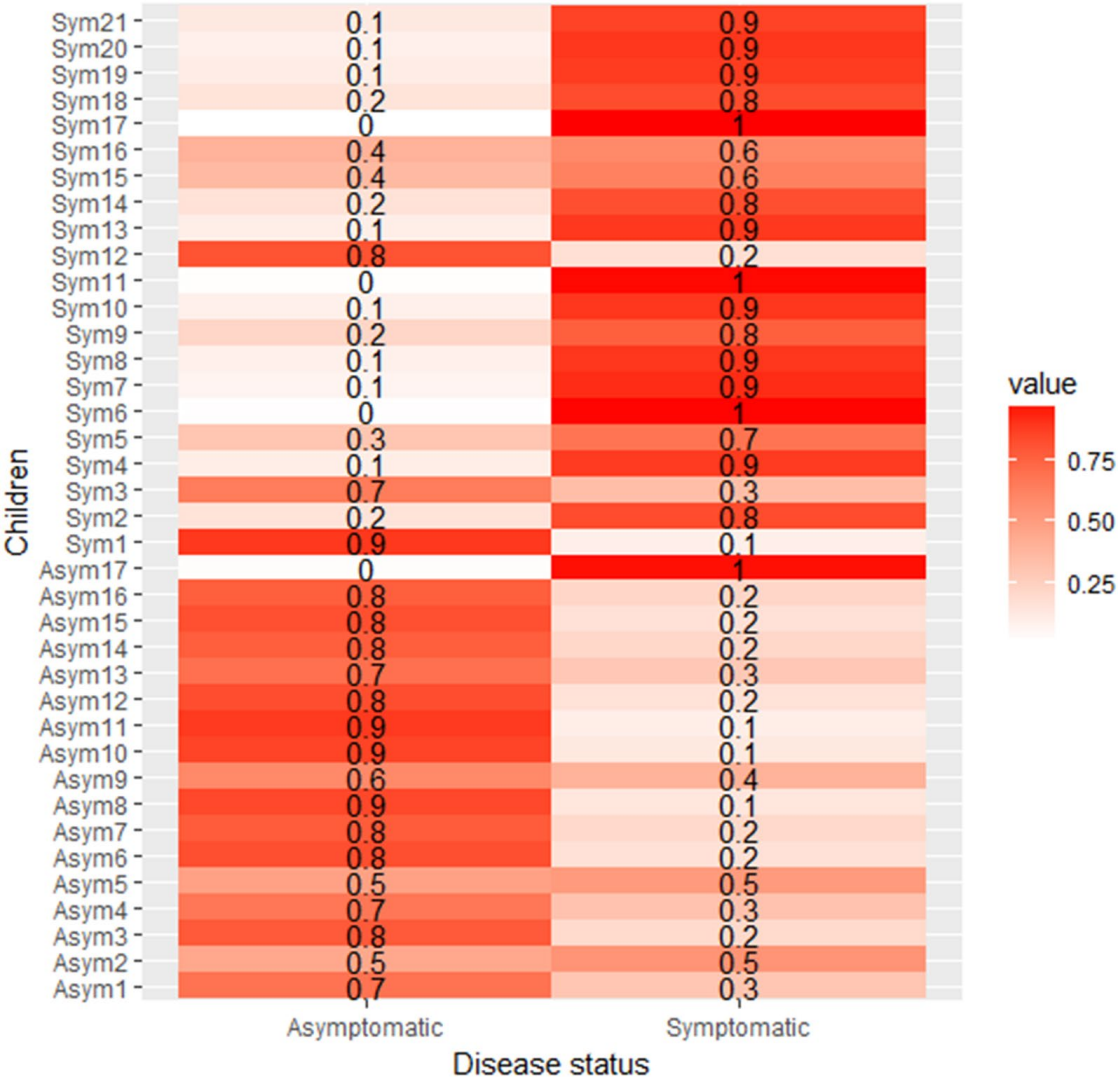
T cell regulatory and activation markers distinguish between asymptomatic and symptomatic *Plasmodium falciparum* infections. It was assessed whether a machine-learning model based on pre-iRBC stimulation levels of CD8+CD69+ and CD8+CD25+CD69+ T cells and post-stimulation levels of CD4+CD25+Foxp3+ and CD8+CD25+Foxp3+ T cells could accurately predict disease status in asymptomatic and symptomatic children. Thirty-eight children had data for all the 4 T cell phenotypes considered. The children were randomly separated into 5 groups. Fixing one group as a test group, we trained a machine-learning model (precisely a support vector machine) on the other 4 groups and then predicted the disease status of children found in the test group. The process was repeated until each of the groups was used exactly once as a test group. The plot shows a representative heatmap of the predicted probability that each child is either asymptomatic or symptomatic. Strikingly, as expected, most asymptomatic (respectively symptomatic) children have a higher predicted probability of being asymptomatic (respectively symptomatic)

Machine learning was used to determine whether the levels of these four T cell phenotypes alone could be used to predict disease status in infected children. A model based on a support vector machine was fitted to the levels of the four T cell phenotypes measured in a sub-set of the infected children and then used to predict disease severity in the remaining children. Using a 5-fold cross-validation analysis to prevent overfitting, it was found that the model accurately distinguishes between asymptomatic and symptomatic children, with a sensitivity of 86%, a specificity of 94%, and an area under the receiver-operator-characteristic curve (AUC) of 90% (Fig. 5). Together, the results suggest that expression levels of the considered regulatory and activation markers determine most of the individual variation in parasitaemia and predict disease status in asymptomatic and symptomatic *P. falciparum* infections.

## Discussion

The aim of this study was to investigate the frequency of activated Tregs and T cell early and late activation markers during *P. falciparum* infections, and the correlations between these and levels of parasitaemia. It was observed that asymptomatic infections are associated with lower levels of Tregs with reduced Treg activation, and reduced expression of T cell activation markers compared to symptomatic infections. Also, T cells from asymptomatic *P. falciparum*-infected children were more responsive to iRBC stimulation compared to cells from symptomatic children. Importantly, the measured variations in regulatory and activation marker levels explained most (68%) of the variation in parasitaemia observed in asymptomatic and symptomatic infections. These results indicate that in contrast to children with symptomatic malaria, there seems to be appropriate levels of immune regulation and activation in children with asymptomatic malaria, which favor the control of parasitaemia. Another, non-mutually exclusive possibility not ruled out by the analyses is that asymptomatic children might have higher levels of protective antibodies compared to symptomatic children, which might contribute to the observed differences in parasitaemia.

Previous data have shown that malaria-exposed individuals can harbour infection without clinical symptoms, implying that there is some level of immune restriction on parasite replication [5]. Increased levels of Tregs have been associated with higher parasitaemia [10, 11] and delayed parasite clearance [20] as well as the development of clinical disease [15, 32, 33]. In this study, it was found that the level of Tregs is higher in children with clinical malaria compared to children with asymptomatic infections and healthy controls. This supports findings from other studies which have also associated increased Treg frequencies with symptomatic malaria infections [9, 23]. Also, the significant increase in the Treg frequency which was observed in the symptomatic children after iRBC stimulation may indicate that during clinical malaria Tregs from the pre-clinical state are expanded or being induced.

The low levels of Tregs observed in the asymptomatic children corroborates the findings of Boyle et al. [32] who identified lower levels of Tregs in children with asymptomatic infections. This was interesting since in another previous study by Jangpatarapongsa et al. [7] they also identified lower amounts of Treg cytokines in individuals with asymptomatic *Plasmodium vivax* infections, suggesting there is less Treg activation in individuals with asymptomatic *Plasmodium* infections. This supports the view that lower levels of Tregs may be associated with a decreased risk of developing clinical disease and possibly an increased likelihood of developing immunity to malaria.

It has recently been shown that Tregs expressing CTLA-4 in murine models of malaria interfere with the acquisition of long-term immunity to malaria infections [13]. The increase in CTLA-4 in Tregs observed in individuals with *P. falciparum* infections compared to uninfected controls could reflect their direct role in controlling immune responses during human malaria infections. Also, the increased expression of CTLA-4 on Tregs in the symptomatic children suggests that immune regulation associated with clinical malaria may affect cellular activation, consequently, affecting the downstream development of anti-malaria immunity.

Importantly, persistent immune activation has been described as a major factor in predicting disease with increased levels of activation being associated with clinical disease progression [34–36]. Resting T cells are identified phenotypically by the absence of CD25/CD69 markers [37]. In this study, activated T cells were classified by the expression of CD25+/CD69+ markers. A significant increase in immune activation in the CD4 and CD8 T cells was observed in clinical malaria. This is in line with a previous study that observed increased immune activation during clinical malaria infections [38]. However, it should be noted that the increased activation in symptomatic children may not directly connote an effective T cell response since cytokine profiles were not measured.

In contrast, in the asymptomatic children, it was found that fewer cells expressed both activation markers on either CD4 or CD8 T cells indicating reduced cellular activation when compared to children with symptomatic malaria. A plausible interpretation of these results is that lower immune suppression by Tregs in asymptomatic children leads to more effector T cell activation and greater parasite control, which in turn feeds back to reduce T cell activation. Conversely, higher immune suppression in symptomatic children might limit parasite control leading to higher levels of parasitaemia and T cell activation. This is in line with the observation that *P. falciparum* activates the immune system in a dose-dependent manner [39]. In addition, the lack of symptoms, lower levels of immune activation, and lower parasitaemia in the asymptomatic children might also result from higher levels of parasite-specific antibodies that reduce parasitaemia levels below the threshold required to induce a T cell response. Additional research is needed to elucidate these hypotheses.

It has been shown that asymptomatic children maintain levels of CD4+CD25+FOXP3- effector T cells that co-produce IFNγ, TNF and IL-10, describing these cells as self-regulatory [9, 40]. Even though lower levels of Tregs and activated Tregs were observed in the asymptomatic children, CD4+CD25+FOXP3− T cells were not significantly different between the asymptomatic and symptomatic children. This may suggest that during asymptomatic malaria, restriction of parasite replication and inflammation may be mediated by these self-regulating effector T cells.

Unfortunately, this study had a number of limitations since a longitudinal study could not be conducted to determine if any of the asymptomatic cohorts may develop clinical disease because they were treated when diagnosed. Consequently, the possibility that the immune dynamics observed reflect changes that occur during the natural course of *P. falciparum* infections could not be ruled out. In addition, because parasitaemia was determined by microscopy, it was not possible to determine conclusively that none of the healthy cohort had sub-microscopic infection. It was also, not possible to evaluate the humoral response in the study population to determine its contribution to the immune dynamics observed.

## Conclusion

The study shows evidence that Tregs are lower and associated with reduced Treg activation in children with asymptomatic *P. falciparum* infections, which corresponds to reduce cellular activation and lower levels of parasitaemia. Also, the greater expansion of activation markers after iRBC stimulation in asymptomatic children compared to symptomatic children suggests that the former children harbour a larger latent repertoire of parasite-responsive T cells. Alternatively, this observation could reflect less Treg-mediated suppression of T cell activation in cells from asymptomatic children. Together, these data support the view that the dynamics of T cell regulation and activation may contribute to the acquisition of anti-parasite and/or anti-disease immunity to malaria. Insights into these dynamics might inform the development of malaria vaccines that induce appropriate levels of cellular activation and regulation as well as optimal control of parasitaemia and disease.

## Additional files

**Additional file 1.** Antibody panel and clones used.

**Additional file 2.** Linear regression analysis to determine the level of variation in parasitaemia using 4 T cell phenotypes.

**Additional file 3.** Diagnostic plots for the regression analysis described in the legend of Additional file 2.

## Abbreviations

Tregs: regulatory T cells
CTLA-4: cytotoxic T-cell lymphocyte antigen 4
PBMC: peripheral blood mononuclear cells
iRBCs: infected red blood cells
uRBCs: uninfected red blood cells
IFNγ: interferon gamma
TNF: tumour necrosis factor
IL-10: interleukin 10
DMSO: dimethyl sulfoxide
