# Supplementary material for: Characterization of T cell activation and regulation in children with asymptomatic Plasmodium falciparum infection
Source: Malar J. 2018 Jul 13;17:263. doi: 10.1186/s12936-018-2410-6 (PMC6045887; doi:10.1186/s12936-018-2410-6)
Supplement: Supplementary file 1 — Additional file 1. Antibody panel and clones used. [file 12936_2018_2410_MOESM1_ESM.docx]

**Additional file 1** List of antibody panels

| **Antibody** | **Clone** |
| --- | --- |
| Anti-CD3 | SK7 |
| Anti-CD4 | SK3 |
| Anti-CD8 | SK1 |
| Anti-CD25 | M-A 251 |
| Anti-CD28 | CD28.2 |
| Anti-CD57 | NK-1 |
| Anti-CD69 | FN50 |
| Anti-CD279 | EH12.1 |
| Anti-CD152 | BN13 |
| Anti-FOXP3 | 206D |
