# Supplementary material for: Characterization of T cell activation and regulation in children with asymptomatic Plasmodium falciparum infection
Source: Malar J. 2018 Jul 13;17:263. doi: 10.1186/s12936-018-2410-6 (PMC6045887; doi:10.1186/s12936-018-2410-6)
Supplement: Supplementary file 2 — Additional file 2. Linear regression analysis to determine the level of variation in parasitaemia using 4 T cell phenotypes. [file 12936_2018_2410_MOESM2_ESM.docx]

**Supplemenatry Data**

**Additional file 1**


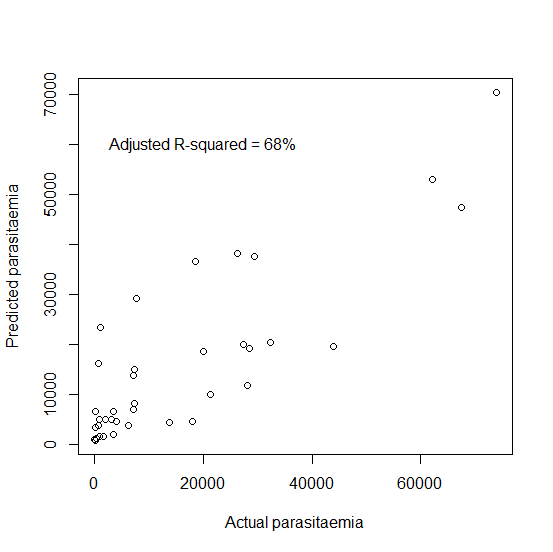


**Additional file 1. T cell activation and regulatory markers govern parasitaemia.** We analyzed a dataset consisting of levels of parasitaemia and of 24 T cell phenotypic markers measured in PBMCs from 57 children, before and after stimulating the PBMCs with *P. falciparum*-infected red blood cells (iRBCs). Two asymptomatic children had no parasitaemia data and were excluded from the analysis. An initial correlation analysis identified significant Spearman’s rank correlations between parasitaemia vs. levels of CD8+CD69+ (r=0.4128658, P=0.0016) and CD8+CD25+CD69+ (*r*=0.4070214, *P*=0.0018) T cells measured before iRBC stimulation, and levels of CD4+CD25+Foxp3+ (*r*=0.4772815, *P*=0.0002) and CD8+CD25+Foxp3+ (*r*=0.4772714, *P*=0.0003) T cells measured after iRBC stimulation. Significance of the correlations was determined using a permutation test. We next performed a linear regression analysis to determine the amount of variation in parasitaemia explained by levels of these four T cell phenotypes in the 41 asymptomatic and symptomatic children. A diagnostic test identified one out of these 41 children to be an outlier (see Additional file 2). After removing this outlying child, levels of the four considered phenotypes were able to explain 68% of the variation in parasitaemia. In the plot, parasitaemia levels measured in the children are shown on the x-axis while the levels predicted by the regression model are shown on the y-axis.
