# Supplementary material for: Characterization of T cell activation and regulation in children with asymptomatic Plasmodium falciparum infection
Source: Malar J. 2018 Jul 13;17:263. doi: 10.1186/s12936-018-2410-6 (PMC6045887; doi:10.1186/s12936-018-2410-6)
Supplement: Supplementary file 3 — Additional file 3. Diagnostic plots for the regression analysis described in the legend of Additional file 2. [file 12936_2018_2410_MOESM3_ESM.docx]

**Supplementary Data**

**Additional file 2**


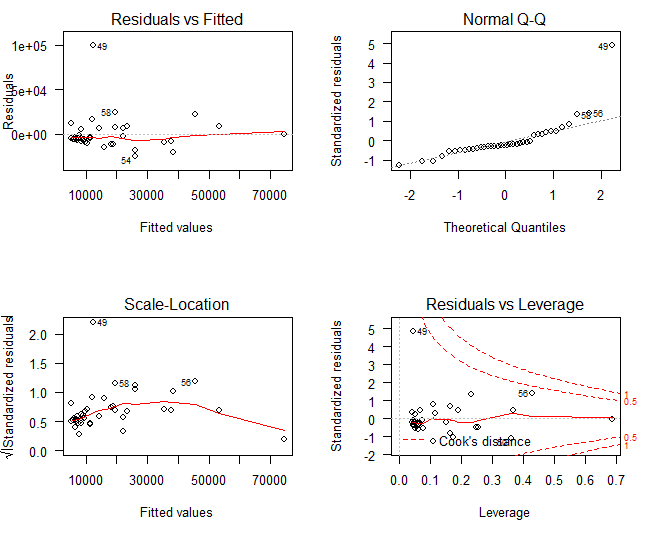


**Additional file 2: Diagnostic plots for the regression analysis described in the legend of Additional file 1.** The plots reveal data points from one of the subjects (subject #49) to be outliers. This subject was excluded from the regression analysis.
